# Tuning-free controller to accurately regulate flow rates in a microfluidic network

**DOI:** 10.1038/srep23273

**Published:** 2016-03-18

**Authors:** Young Jin Heo, Junsu Kang, Min Jun Kim, Wan Kyun Chung

**Affiliations:** 1 Pohang University of Science and Technology (POSTECH), Mechanical engineering, Pohang, 790-784, South Korea

## Abstract

We describe a control algorithm that can improve accuracy and stability of flow regulation in a microfluidic network that uses a conventional pressure pump system. The algorithm enables simultaneous and independent control of fluid flows in multiple micro-channels of a microfluidic network, but does not require any model parameters or tuning process. We investigate robustness and optimality of the proposed control algorithm and those are verified by simulations and experiments. In addition, the control algorithm is compared with a conventional PID controller to show that the proposed control algorithm resolves critical problems induced by the PID control. The capability of the control algorithm can be used not only in high-precision flow regulation in the presence of disturbance, but in some useful functions for lab-on-a-chip devices such as regulation of volumetric flow rate, interface position control of two laminar flows, valveless flow switching, droplet generation and particle manipulation. We demonstrate those functions and also suggest further potential biological applications which can be accomplished by the proposed control framework.

Accurate and stable flow control in microfluidic channels is a fundamental requirement in lab-on-a-chip devices to investigate biological systems^1^,^2^,^3^. Control of flow in a micro-channel is required for precise manipulation of a sample solution or reagent, and for stimulation of cells by mechanical shear force^4^. Many studies have focused on developing miniaturized valves and pumps to dynamically regulate flow in micro-channels^5^,^6^. However, those miniaturized systems do not provide reliable accuracy and precision; especially, mechanical valves or pumps use moving parts, which are often unreliable and can cause failure of accurate flow control^7^.

A flow source such as a syringe pump is typically used to generate a constant flow from the external macro-environment into a micro-channel. However, when a syringe pump is applied to a micro-channel, flow fluctuates due to the motion of the electric motor and deformation of elastic channel walls^8^,^9^,^10^. This fluctuation is unavoidable and causes inaccuracy and instability in flow regulation. A pressure source such as a pressure pump is also widely used to drive flow more stably with faster responses than those of the flow source. However, accurate control of the flow with the pressure source is difficult to achieve in the presence of parametric uncertainties or disturbances in a microfluidic network^11^. For example, fabrication error, air bubbles in the channel, wobble of tubes, suspended cells, and the swelling of the channel can degrade the accuracy of flow regulation^12^.

For this reason, some lab-on-a-chip devices adopt a feedback control scheme to achieve high precision in flow control. A feedback control scheme is suitable for complex networks and does not require exact model parameters (e.g., fluidic resistances or capacitances). Most research into feedback control schemes for microfluidic devices and commercialized pressure pump systems have only considered a proportional-integral-derivative (PID) controller^13^,^14^,^15^, which is insensitive to disturbance and has good control accuracy. However, a standard PID controller cannot be easily applied to a complex microfluidic network because a microfluidic network is a coupled multiple-input multiple-output (MIMO) system. Furthermore a PID controller requires a tedious gain-tuning process which affects control accuracy and is difficult to conduct for users who are not familiar with control theory. To the best of the authors’ knowledge, a control scheme for a complex microfluidic network has not been developed.

In this study, for the first time, we propose a robust control algorithm to accurately regulate flow in a microfluidic network in the presence of external disturbances and model uncertainties while maintaining stable steady-state flow. The proposed control algorithm can be broadly applied to microfluidic systems for accurate and precise flow regulation, and does not require a tedious gain-tuning process. We theoretically analyzed and experimentally validated the performances of the proposed control algorithm including the robustness, the optimality and the stability of the closed-loop system. We also demonstrated some useful applications for lab-on-a-chip devices that can use the proposed robust controller: regulation of volumetric flow rate, valveless flow switching, interface position control of two laminar flows, fine droplet generation, and particle manipulation.

## Control Framework

### Fluidic circuit for modeling microfluidic network systems

**Figure 1:**
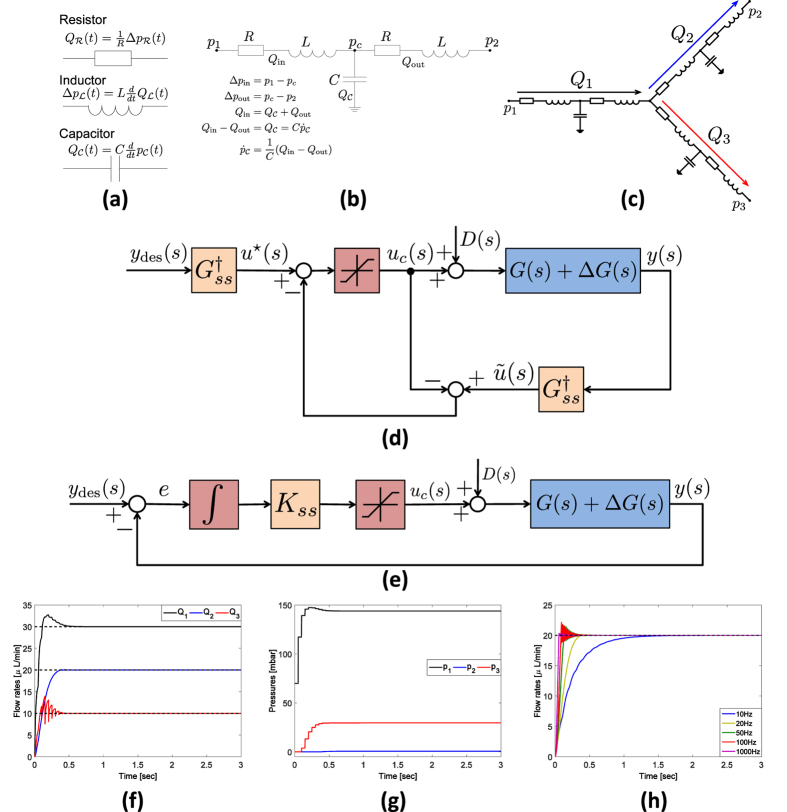
Microfluidic network modeled by fluidic circuit components and robust control framework. (**a**) *RLC* components for a fluidic circuit. The resistor and inductor can be used to represent laminar incompressible flow 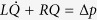. The capacitor represents compliance effects of channel walls and tubes. (**b**) T-shaped model that represents fluid flow in a single microfluidic channel. Two resistors and two inductors are connected with a single capacitor. (**c**) A Y-junction network constructed by synthesizing three T-models. This network will be used in simulations and experiments to verify performances of the proposed robust controller. (**d**) The structure of the proposed robust control framework. Undesirable effects and perturbation will be removed by means of structure. (**e**) The equivalent structure of (**d**). (**f**) Output flow rates of the numerical simulation. The robust controller is applied to the Y-junction network in (**c**). The steady-state error is zero in the presence of parametric uncertainties. (**g**) Corresponding input pressures of the numerical simulation. (**h**) Simulation results with respect to varying control frequencies. The steady-state error is zero and the transient responses become faster as the control frequency gets higher.

To apply control theory and numerical simulations for analyzing the proposed controller, a dynamic model of fluid flow in a microfluidic network should be constructed. The laminar incompressible flow in a single channel can be modeled by a fluidic resistance *R* and a fluidic inductance *L* as 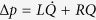 where Δ*p* is a pressure drop between the ends of the single channel, and *Q* is volumetric flow rate. The compliance of the channel wall, compressibility of fluid, and effects of air bubbles in the microfluidic channel are represented by a fluidic capacitance *C* as 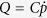 (Fig. 1a). Using the three *RLC* components, we construct a model of a single microfluidic channel that has T-shaped geometry composed of two resistors, two inductors and a single capacitor (Fig. 1b). An arbitrary microfluidic network can be modeled by connecting several T-models (Fig. 1c) and represented by a coupled MIMO system. Here, it is worth noting that transient responses (flow rates) with respect to applied pressures of the network model rely on *R, L, C* and the network topology, whereas steady-state responses are only affected by the resistances and the network topology.

We define a *perturbed system* as *G*(*s*) + Δ*G*(*s*) where *G*(*s*) is the transfer function matrix of an unperturbed microfluidic network composed of *RLC*s (*nominal system*) and Δ*G*(*s*) is *unknown perturbations* such as unmeasurable fabrication error and the uncertain elasticity of tubes. We also define the *steady-state gain G*_*ss*_ that is composed of only nominal resistances by removing the inductors and the capacitors from the nominal system *G*(*s*); i.e. *G*_*ss*_ = *G*(*s*)|_*s*=0_ (see Methods for detailed construction method of the steady-state gain). The steady-state gain will be used to implement the proposed controller, whereas the perturbed system will be used to analyze the proposed controller and to conduct numerical simulations.

### Robust control algorithm

The main objectives of the proposed controller are to eliminate perturbations and to minimize input pressure while guaranteeing stability of the closed-loop system; i.e., the closed-loop system is insensitive to model uncertainties Δ*G*(*s*) (e.g., unknown resistances, capacitances, and inductances) and disturbances *D*(*s*) (e.g., flow agitation in tubes connected from a pressure pump to a microfluidic chip) while maintaining its high-accuracy flow regulation. The input vector 
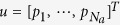
 consists of *N*_*a*_ pressures at each input port and the output vector 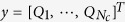 consists of *N*_*c*_ flow rates at each channel. The controller only uses a right inverse of the steady-state gain *G*_*ss*_ to force the steady-state flow of the perturbed system *G*(*s*) + Δ*G*(*s*) to become desired flow rate 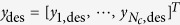 (Fig. 1d); hence, the proposed controller can be implemented without knowledge of the exact values of resistances, capacitances, and inductances.

The fact that the controller compensates for all undesirable effects at steady-state can be proved by computing a block diagram algebra of the control structure (Fig. 1d) as


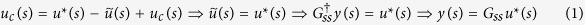



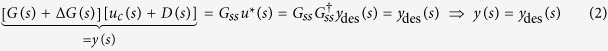


where the superscript † represents the right inverse of the matrix (i.e., *A*^†^ = *A*^*T*^(*AA*^*T*^)^−1^). Here, *G*_*ss*_ maps reference input u^*^(*s*) to desired output *y*_des_(*s*) and the system output *y*(*s*) becomes the desired output *y*_des_(*s*) by the control input *u*_*c*_(*s*). Hence, the controller makes the perturbed system *G*(*s*) + Δ*G*(*s*) converge to the desired output in the presence of disturbances *D*(*s*).

From the control structure (Fig. 1d), discrete control input to implement the controller in a microfluidic system is derived as


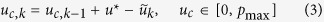



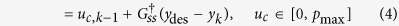



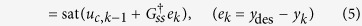


where the subscript *k* is the time-step index, *y*_*k*_ is output measurement at step *k*, and 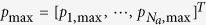 represents the maximum pressure at the input ports that does not saturate the pressure pump. The saturation function in (5) is defined by


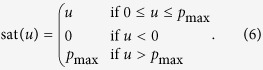


It is interesting to note that the control structure can be converted to an integral controller (Fig. 1e). The derivation of the conversion is as follows:


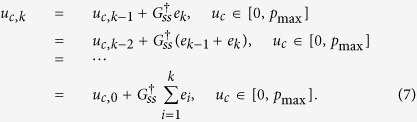


Here, if we assume that the initial control input *u*_*c*,0_ is zero, then the resulting discrete control input is


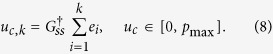


Therefore, the resulting control law is nothing but the integral control because (8) can be expressed as 
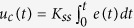
 where 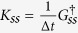 and Δ*t* is time interval (Fig. 1e); in short, the structure of the controller can be interpreted as integral action with gain *K*_*ss*_. Whereas the conventional integral action in a PID controller has an integral gain which is a constant diagonal matrix, the gain 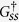 (or *K*_*ss*_) in our controller optimally distributes the error signals to the multiple input sources (as will be discussed in following section). The resulting closed-loop system is always stable because the closed-loop system is just the second order system cascaded by integral control. Please refer Supplementary Information Text S1 for the detailed stability analysis.

### Optimality of control input and tuning-free property

The steady-state gain matrix *G*_*ss*_ is a linear transformation from given input pressure *u* to output steady-state flow *y*. Physically, pressure applied to a microfluidic chip from several pressure sources generates the pressure drops across the microfluidic network, then the given pressure drops produce resultant steady-state flow in channels. Hence, the steady-state gain *G*_*ss*_ is only composed of fluidic resistances and network topology because steady-state flow depends entirely on the fluidic resistances of the network. Mathematically, the steady-state gain *G*_*ss*_ is an *onto matrix* and always has a right inverse matrix (see Supplementary Information Text S1 for the proof of existence of a right inverse matrix). A right inverse can be used to find a least-norm solution as *u*_ln_ = *A*^*T*^(*AA*^*T*^)^−1^*y* = *A*^†^*y* where *u*_ln_ is the optimal solution of *y* = *Au* that minimizes 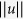; i.e., *u*_ln_ is a solution of the optimization problem as follows


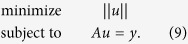


Here, for the case of the proposed control input (5) or (8), *A* = *G*_*ss*_ and *y* = *y*_des_ − *y*_*k*_(=*e*_*k*_) in (9). This means that the control gain 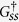 yields the same effect as using the simple closed-form in (5) or (8) to solve the optimization problem (9) at every time interval. This optimality implies that the pressure source requires only the minimum pressure whereas a PID controller does not optimize control input, and therefore wastes pump capability.

Moreover, due to the optimal property, the proposed controller yields a tuning-free property that is its key characteristic. The tuning-free property enables easy implementation of the proposed controller to a complicated microfluidic network without tedious and technical gain tuning process. For example, using a typical PID control scheme to simultaneously control several flows in a microfluidic network, the number of gains that should be finely tuned increases in proportion to the number of controllable channels; if gains that should be tuned >10, it is impractical to be implemented. In addition, we provide a single tuning parameter to adjust the closed-loop transient response that can be used when fast response is required (Supplementary Information Text S2).

### Simulation

Numerical simulations were performed to verify the robustness, optimality, stability and accuracy of the proposed controller. As a network model for the simulation, a simple Y-junction network in which three channels are connected to each other was used (Fig. 1c), and unknown parameter uncertainties were included in the network model. The control algorithm only used the steady-state gain which consists of nominal resistances of channels and the network topology, although the capacitance, inductance and uncertain parameters affect the resulting flow.

Set point regulation using the controller was performed with desired flow rates in each channel set to 10, 20 and 30 *μ*L/min, respectively. The output flow rates converged to the desired flow rates quickly (<1 sec) without steady-state error, whereas the redundant pressure *p*_2_ remained at zero due to the optimality of the control input (Fig. 1f,g). Simulations were also performed at varying control frequencies (Fig. 1h). As the control frequency increased, the response time decreased, but small oscillations occurred in the transient response if the control frequency was >20 Hz. This is because the control frequency scales control gain (see Supplementary Information Text S1 for a relationship between the control frequency and the system stability). The control frequency affected only transient responses; the steady-state responses were the same at all frequencies. This means that the flow regulation accuracy is guaranteed at a wide range of control frequencies and that the transient response can be adjusted by a single tuning parameter (Supplementary Information Text S2).

## Results

**Figure 2:**
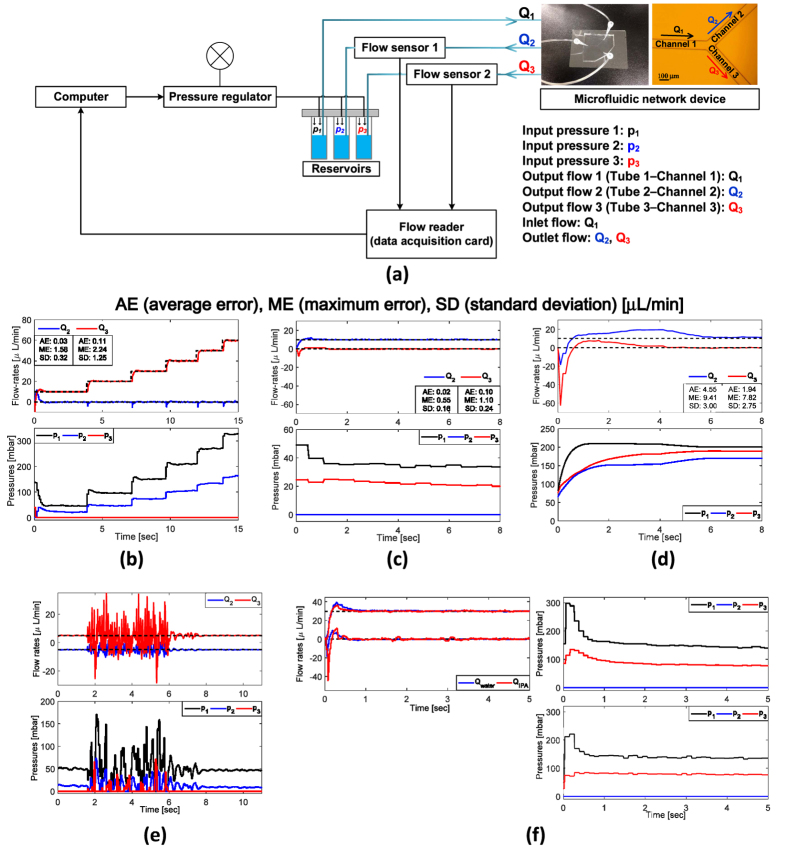
Experimental results to verify performances of the tuning-free robust controller. (**a**) The experimental setup for flow regulation using the proposed robust controller. The flow sensors measure flow signal (*Q*_2_, *Q*_3_) and the computer sends the control signal (*p*_1_, *p*_2_, *p*_3_) to the pressure regulator in real time. (**b**) Output flow rates of flow regulation (top) and its corresponding input pressures (bottom). The average steady-state error is 0.03 *μ*L/min (maximum error: 1.58 *μ*L/min, standard deviation: 0.32 *μ*L/min). (**c**,**d**) Experimental results of a well-tuned PID controller and the proposed robust controller, respectively. Output flow of the proposed control shows faster and more stable responses than the PID controller. All input pressures of the proposed controller are less than those of the PID controller. (**e**) Experimental result of flow regulation in the presence of tube wobble to verify the robustness against to disturbances. The jiggling had been applied for 4 s. After the jiggling ceased, flow rate was instantaneously stabilized. (**f**) Experimental result of flow regulation to verify the robustness against to parameter uncertainty. Two experiments were performed with same conditions except for the working fluid (water and IPA). Steady-state responses of two experiments are almost the same regardless of fluid (left top). Right top and bottom depict the applied pressure input in case of water and IPA, respectively.

We conducted experiments in a microfluidic chip to confirm the characteristics of the proposed controller. The experimental setup is composed of the computer, pressure regulator, reservoirs, microfluidic device, flow sensors and flow reader (Fig. 2a). Using the pressure regulator, pressure is independently applied to the reservoirs to force the fluids into the microfluidic chip through tubes. Two flow rates (*Q*_2_, *Q*_3_) are measured using independent flow sensors, one located at each of the tubes that connect the reservoirs to the microfluidic device. The flow reader sends the measured flow signal to the computer, which sends control signals to the pressure regulator. Using the experimental setup, basic flow-regulation experiments were performed to validate the robustness, optimality and accuracy of the proposed controller. In addition, a well-tuned PID controller was also tested for comparison with the robust controller. A Y-junction microfluidic network, which is the same model used in the simulations, was connected to three pressure sources. Three pressure input ports *p*_1_, *p*_2_, *p*_3_ were located one at the end of each channel. Flow rates *Q*_1_, *Q*_2_, *Q*_3_ of each channel are the outputs to be controlled, but only two flow rates *Q*_2_ and *Q*_3_ were measured by flow sensors in the experiments (see Supplementary Information Text S1 for the selection method of controllable channels).

Moreover, some lab-on-a-chip applications based on the basic flow control were also demonstrated to show versatile functions of the robust feedback control system. A video clip of those experiments is provided as Supplementary Information.

### Basic flow regulation

Basic flow regulation experiments were performed to show that the proposed controller is robust against geometrical uncertainty and disturbances while maintaining accurate flow regulation with minimum pressure. In set point regulation experiments (Fig. 2b), desired flow rate in channel 3 was changed manually from 10 *μ*L/min to 60 *μ*L/min while flow rate in channel 2 remained zero. The output flow rate shows fast transient responses with rise times less than 0.5 s in every case and stable steady-state responses with high-accuracy in the presence of unmeasurable fabrication error; i.e., the control performance is insensitive to fabrication quality (Fig. 2, top of b). Moreover, the pressure input was minimally used due to the optimality of the proposed control algorithm as we mentioned in the previous section; one redundant pressure source *p*_3_ remained zero (Fig. 2, bottom of b).

### Comparison with PID control

The proposed robust controller was compared with a conventional PID control scheme to show the better regulation performance and advantages of the tuning-free property (Fig. 2c,d). Average error of the proposed controller is <0.1*μ*L/min, which is much less than the PID result of 1.94 to 4.55 *μ*L/min; the maximum error of the proposed controller is less than 1/7 times that of PID control. Control input (i.e., pressure applied to the microfluidic chip) also shows the advantage of the proposed controller. The proposed controller required pressure input less than one fifth of those that the PID controller required. This optimality of the pressure input is appropriate for microfluidic chips in which high pressure cannot be easily applied^16^.

### Robustness to the disturbances and fluid property

The presence of tube wobble is a usual event during use of a microfluidic device. To show the algorithm’s insensitivity to these disturbances, we intentionally jiggled the tubes connected from the pressure pump to the microfluidic chip while operating the control algorithm. The flow rate signals oscillated during the jiggling, but the oscillation was maintaining its center near the desired flow rates and the flow rate converged instantaneously to the desired steady-state after the jiggling ceased (Fig. 2e).

The proposed controller is also insensitive to change of fluid properties (e.g., viscosity). To show the robustness to fluid variations, different fluids – water and isopropyl alcohol (IPA) – were used for flow regulation experiments. Although input pressures and transient responses with IPA were somewhat different from those of water, the comprehensive regulation accuracy was almost the same (Fig. 2f). The proposed controller does not need gain tuning due to its tuning-free property, whereas conventional control methods need control gain modification that depends on the fluid dynamics.

These two experiments have confirmed that the proposed controller is insensitive to both external disturbances *D*(*s*) and internal parametric uncertainties Δ*G*(*s*). This robustness is appropriate to applications that require long-term stability and high precision, e.g., cell culture in a lab-on-a-chip device in the presence of slowly-varying dynamics such as effects of absorption into poly(dimethylsiloxane) (PDMS)^4^,^17^.

### Versatile applications

Many lab-on-a-chip applications such as flow interface position control^15^,^18^,^19^, valveless flow switching^16^,^20^,^21^,^22^, droplet generation^14^,^23^ and particle manipulation can be easily realized using the proposed flow control method. In this study, those were implemented to show the versatility of the proposed flow controller in the Y-junction network.

**Figure 3:**
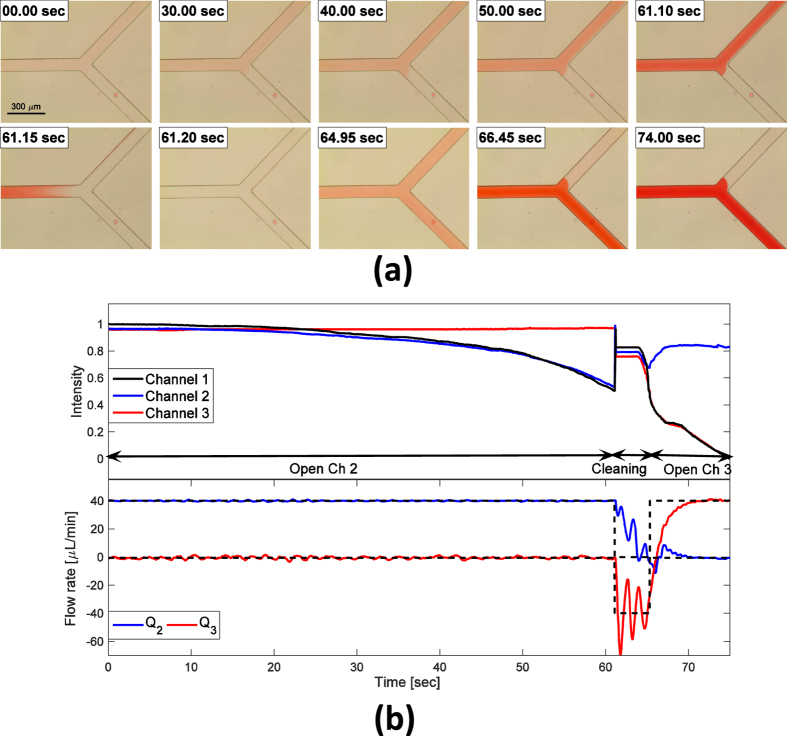
Valveless flow switching experiment. (**a**) Image sequence recorded during the experiment. (**b**) Intensity in each channel with respect to time during the experiment (top) and actual flow rates in channels 2 and 3 with desired flow rates of each channel (bottom).

In the valveless flow switching experiment (Fig. 3), a dye solution and DI (deionized) water were used to visualize the flow. The experimental result shows a switching of the dye solution at a fork in the Y-junction network by simultaneously controlling both upper and lower flows (*Q*_2_ and *Q*_3_). During the ‘Open Ch 2’ stage, the upper channel was open and the lower channel was closed, both for 61 s. The reference flow rate of the open channel was set to 40 *μ*L/min and the flow rate of the closed channel was set to 0 *μ*L/min. At this time, intensities (concentrations) at the centers of channels 1 and 2 both decreased (concentration increased) while intensity at the center of channel 3 was maintained (top of Fig. 3b); as a result the solution darkened. At the same time, flow rates in channels 2 and 3 were maintained at their reference flow rates (bottom of Fig. 3b). From 61.15 s to 65.3 s channels were cleaned to remove the dye solution, then the opposite side channel (channel 3) was opened to execute a valve switching. In this stage, intensities decreased in channels 1 and 3, but remained the same in channel 2. Also the flow rates in both channel 2 and channel 3 converge to the reference flow rates as in the previous step. The method to compute flow intensity is described in Supplementary Information Text S3.

**Figure 4:**
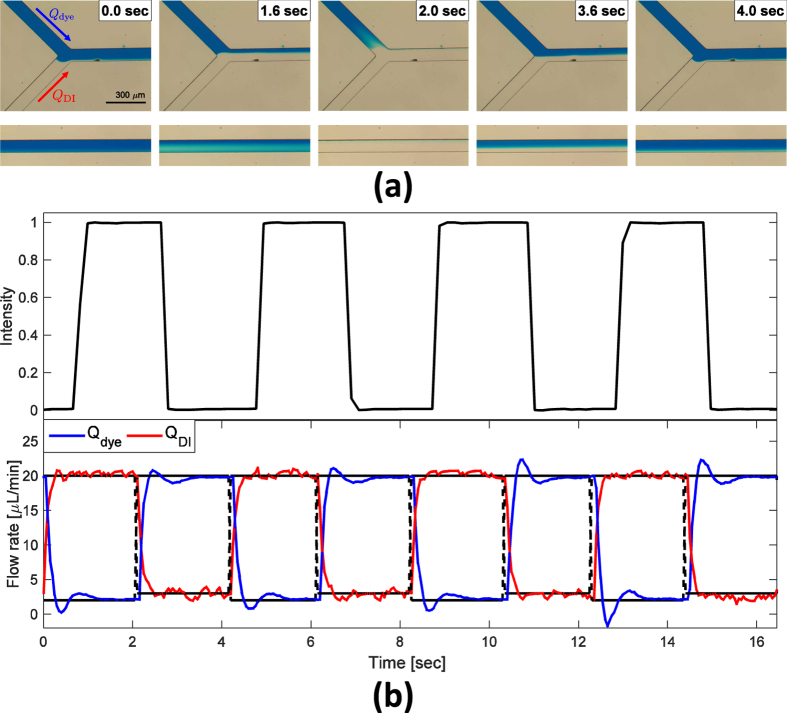
Control of laminar interface position. Reference trajectory is square-wave that has 0.25 Hz along the channel width. (**a**) Image sequence recorded at the junction (top) and downstream (bottom). (**b**) Intensity in the downstream channel with respect to time during the experiment (top) and actual flow rates of *Q*_dye_ and *Q*_DI_ with desired flow rates of them (bottom).

In the laminar interface position control experiment (Fig. 4), two laminar flows (dye solution; DI water) form a flow interface at the confluent channel; the position of the interface can be manipulated by controlling each flow (*Q*_dye_ and *Q*_DI_). Interface position was controlled to track a 0.25-Hz square-wave reference trajectory along the channel width. This trajectory tracking can confirm the precise controllability of interface position in a microfluidic network. The experimental result shows that interface position tracks the reference trajectory well at both the junction and downstream (Fig. 4a). The corresponding intensity at the center of the downstream flow also behaves like the reference square-wave (Fig. 4, top of b). At the same time, flow rates of each channel also successfully tracked the reference flows (Fig. 4, bottom of b).

**Figure 5:**
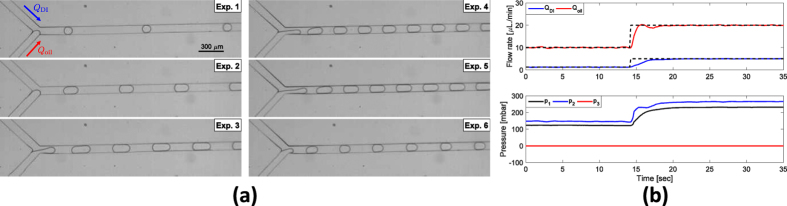
Droplet generation experiment. (**a**) Image sequence recorded during each experiment. (**b**) Flow rate response when the desired flow rate changed from Experiment 1 to Experiment 6 (top) and its corresponding input pressures (bottom).

In the droplet-generation experiment (Fig. 5), silicone oil-in-water emulsion was finely generated using the flow control. The size of droplets and the generation frequency are tunable by controlling flow rates of dispersed and continuous phases in two channels (*Q*_DI_ and *Q*_oil_). Six sets of experiments were performed by changing flow rates of water phase and oil phase, respectively (Supplementary Information Text S4, Table S1). We used a high-speed camera to record microscopic images at 500 fps for 4 s during each experiment. We measured the major axis length of droplets in each combination of water and oil flowrates. The size distribution of the droplets was very narrow; in the worst case, s.d./mean was ~0.04; this means that fine droplets can be generated consistently in the presence of fluctuating interface and the existence of droplets in the channel. Moreover, the experiment sets follow a scaling law that describes droplet size with respect to flow rates in a T-junction^23^. In addition, when the desired flows were changed from Experiment 1 to Experiment 6, flow rates smoothly converged to the desired flow rates (Fig. 5b). Supplementary Information Text S4 provides detailed analysis of droplet size with respect to flow rates, and describes an image-processing algorithm to compute size of droplets.

**Figure 6:**
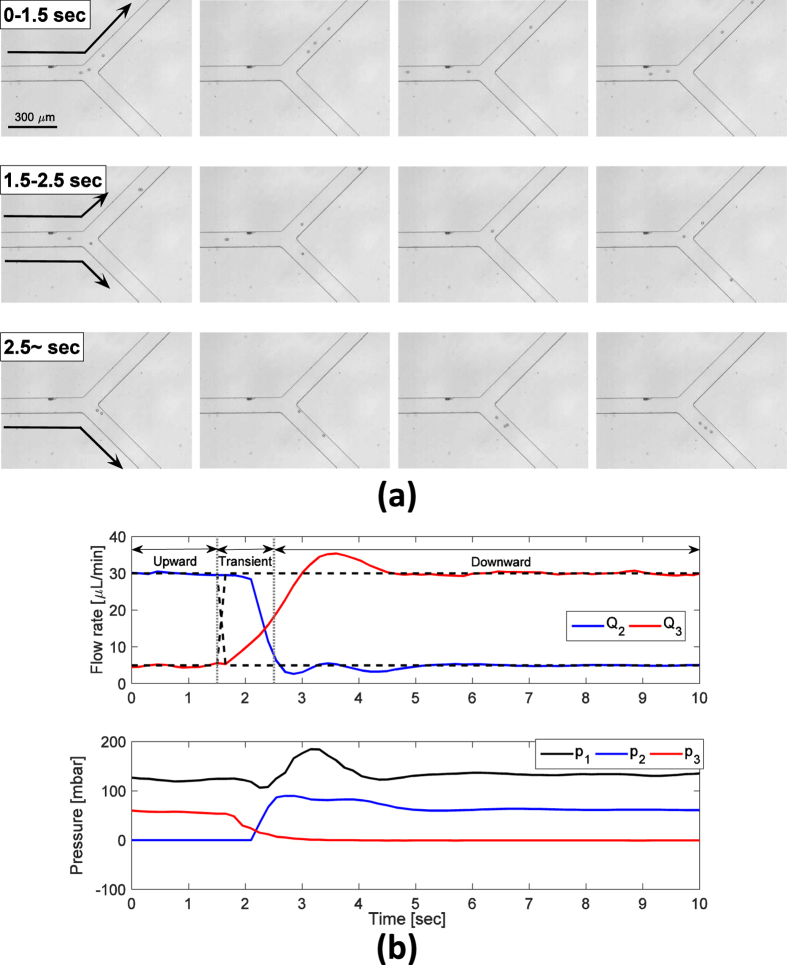
Particle switching experiment. (**a**) Image sequence recorded during the experiment. (**b**) Flow rates in channels 2 and 3 with respect to time during the experiment (top) and its corresponding input pressures (bottom).

In the particle-switching experiment (Fig. 6), suspensions of spherical polystyrene particles with 15-*μ*m diameter were loaded in the microfluidic chip. At the beginning, desired flow rates at channel 2 and 3 were set to 30 *μ*L/min and 5 *μ*L/min, respectively. Due to the dominance of convective flow to channel 2, particles were transported to that channel (upward). The flow direction switched at 1.5 s, at which time the transient started. After the transient region, particles were transported downward. The dominant factor that determines the motion of particles is the drag force of fluid flow, so particles suspended in the fluid can be manipulated by controlling the flow direction. Image sequences and its flow responses show this process well (Fig. 6).

These experiments could be conducted by a pressure pump without the controller, but the controlled system enables consistently stable and accurate responses in the presence of disturbances and parametric uncertainties while maintaining the optimal pressures. Therefore, by applying the proposed controller, the accuracy and stability of these applications can be increased regardless of what sort of a microfluidic chip it uses.

## Discussion

Lab-on-a-chip technologies have enabled both high-fidelity and high-throughput investigations of biological systems. Accurate and precise regulation of microenvironments for experiment has become increasingly important as the technologies have been expanded^24^. Direct control of flow from external pressure sources is an obvious method to change flow dynamically and temporarily, but was difficult to use due to lack of a control strategy for complex networks^11^. In this study, we provided a robust control algorithm which is applicable to complex microfluidic networks. There are many advantages in using the flow control based on pressure sources. Firstly, flow induced by a pressure source stabilizes quickly to steady-state, whereas a flow induced by a flow source (syringe pump) stabilizes slowly. Secondly, an independent pressure sources connected to inlet ports can simultaneously control multiple-channels. Those fast responsiveness and simultaneous controllability enable dynamic flow change that can displace an on-chip valve or pump (such as the Quake group’s on-chip pneumatic valve^25^). Thirdly, the system is applicable to rigid microfluidic chips such as thermoplastic or glass because pressure is applied from external sources. For the case of the on-chip valve, a rigid microfluidic chip cannot be used because most of on-chip valves and pumps are fabricated by multi-layer soft lithography. This absence of a valve function for a rigid chip has troubled the commercialization of lab-on-a-chip devices.

We also compared the proposed control algorithm with a conventional PID control scheme for flow control by pressure sources. A well-tuned PID control may have better responses than the proposed method but requires tedious and technical tuning for many gains. For a Y-junction network that has three channels (two of them controllable), each gain (P, I, D) with respect to each channel (channel 2, 3) should be tuned; i.e., the number of tuning parameters is 3 × 2 = 6 and it is hard to be finely tuned in practice. If a complex network has >10 channels, the number of tuning parameters is >30. In this case, tuning is not practical; this is the main disadvantage of using a PID controller for a microfluidic network. In contrast, the proposed controller provides robustness and optimality of control input without such an impractical tuning process.

The controller also guarantees robustness to perturbations. By applying the controller, the system becomes *insensitive* to geometrical uncertainties, fluid properties and external disturbances; it enables high-accuracy and high-precision flow regulation regardless of variations, such as cross-sectional shape of the channel, fabrication quality, fluid property, and the presence of environmental fluctuation. The insensitivity to the fluid properties means that users do not need to know the exact fluid properties such as fluid viscosity or fluidic capacitances. The insensitivity to external disturbances empowers the controller to compensate for undesirable effects such as the tube wobble that commonly occur during experiments; thus, the controller improves the stability of microfluidic experiments which are sensitive to the cellular microenvironment. In all regulation experiments performed in the previous section, average steady-state error was <0.02 *μ*L/min and response time was <1 s.

To apply the proposed feedback control scheme, a measurement must be provided to the controller at every time interval. Any flow sensor that can reliably measure flow rate can be used in the feedback control system. We used a commercial calorimetric low-flow liquid flow meter that estimates flow rate by measuring temperature profile at different two points. This flow meter has nanoliter per minute resolution and is appropriate for use in microfluidic devices, but some limitations occur in particle experiments due to accumulation of particles in the sensor capillary. Therefore, to ensure that the flow rate feedback is accurate, the device must be cleaned after use to prevent deposition of solids. In general, closed-loop responses depend on the resolution of a sensor, so its accuracy must be checked before applying the feedback control.

In conclusion, we focused on the development of a robust control algorithm that is easy to use to any researchers for interdisciplinary study. The accurate and precise flow control has many potential applications in lab-on-a-chip devices. For example, control of pH concentration that is important to regulate cellular environments can be accomplished by the proposed robust flow controller^24^,^26^,^27^,^28^,^29^. In addition, the cellular environment can also be regulated in time and space by controlling flow over adherent cells^4^.

## Methods

### Construction of the steady-state gain

The steady-state gain matrix *G*_*ss*_ is composed of only the network resistances because it only considers steady-state flow without dynamic motion of the fluid flow; this means that the function of *G*_*ss*_ is to map input pressures to output steady-state flows. If flow rate vector is 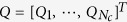 and pressure vector is 
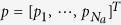
, then *Q* = *G*_*ss*_*p* represents the mapping of pressures to flow rates. *G*_*ss*_ can be obtained by setting *s* to zero in the full nominal *RLC* transfer function *G*(*s*) (*G*_*ss*_ = *G*(0)). However, this approach is a tedious process, and is impractical for use to obtain a full system model. Therefore we propose an easier method to construct the steady-state gain by considering an alternative network that consists of only resistances (*resistance network*).

A resistance network is composed of *N*_*c*_ channels, *N*_*n*_ nodes, and *N*_*a*_ input ports. The continuity condition (Kirchhoff’s current law) can be represented as


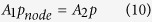


where 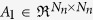 and 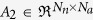 are constant matrices which represent the relationship of node pressures 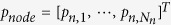 and pressures at input ports 
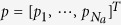
. The pressure drops in the network is represented as


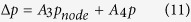


where the pressure drop vector 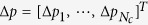 is composed of pressure drops of each channel, and 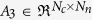 and 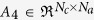 are constant matrices. Substituting (10) into (11) yields


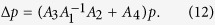


Finally, the steady-state gain matrix can be obtained by Poiseuille law as follows:


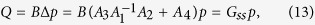



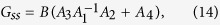


where 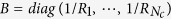 is a *conductance* (reciprocal of a resistance) *matrix* and the matrix 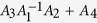 is a *network topology matrix* that can be obtained using the relationships (10) and (11). The steady-state gain is the product of a conductance matrix and a topology matrix (14). Using this alternative method, the steady-state gain can be easily constructed. This construction method does not require any exact parameters (e.g., resistances, inductances, capacitances); it requires only nominal resistances.

As an example, consider the Y-junction network used in the Simulation section. *R*_1_, *R*_2_, and *R*_3_ are the nominal resistances of each channel, *p*_1_, *p*_2_, *p*_3_ are pressure sources at the input ports, and *p*_*n*,1_ is pressure at the node. Using (10) we can obtain


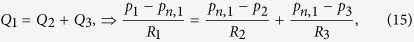



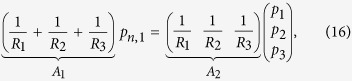


and, using (11) we can also obtain


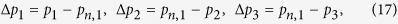



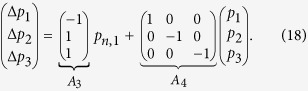


Finally, the steady-state gain matrix can be computed as


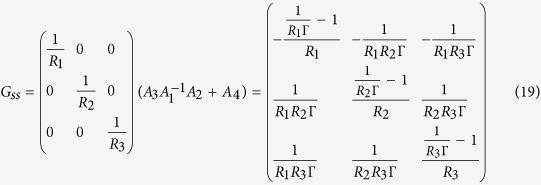


where 
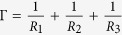
. A more-complex example is described in the Supplementary Information Text S1.

### Fabrication of the microfluidic chips

A Y-junction channel was fabricated using soft lithography^30^. A 50-*μ*m-thick negative photoresist (KMPR 1025, Microchem, USA) pattern was prepared on a 4-inch silicon wafer using conventional photo-lithography to be used as a mold for the micro-channel structure. The PDMS base and curing agent (Sylgard 184 A/B, Dow Corning, USA) were mixed at 10:1 ratio, degassed, cast on the photoresist mold and cured at 100 °C for 15 min. After curing, the PDMS channel was detached from the mold and a biopsy punch was used to create access holes as the inlets and outlets of the channel. The PDMS channel was then bonded onto a slide glass using oxygen plasma treatment. Finally, the access holes were connected to external tubing (Tygon ND 100-80, Saint-Gobain, France) with pipette tips (T-300, Axygen Scientific, USA). Deionized water, isopropyl alcohol and silicone oil (OS-30, Dow corning, USA) were used as the controlled fluid and 15 *μ*m spherical particles (Sigma-Aldrich, USA) diluted in deionized water were used in the particle-flow switching experiment.

### Implementation of the controller

The external system to implement the proposed control algorithm consists of a pressure regulator and flow sensor (MFCS-EZ, Fluigent, France). The pressure regulator applies desired input pressures to sample reservoirs, which are connected to inlets and outlets of the micro-channel. The flow sensor was placed in the middle of connection tubes to measure the flow rates in them. The measured flow rate was sent to a computer and used as a feedback signal. The computer used the control algorithm to calculate the control input and sent it to the pressure regulator in real time. See Supplementary Information Text S1 for the implementation pseudo-code.

## Additional Information

**How to cite this article**: Heo, Y. J. *et al*. Tuning-free controller to accurately regulate flow rates in a microfluidic network. *Sci. Rep.* **6**, 23273; doi: 10.1038/srep23273 (2016).

## Supplementary Material

Supplementary Information

Supplementary Video S1
