# Supplementary material for: Tuning-free controller to accurately regulate flow rates in a microfluidic network
Source: Sci Rep. 2016 Mar 18;6:23273. doi: 10.1038/srep23273 (PMC4796872; doi:10.1038/srep23273)
Supplement: Supplementary Information [file srep23273-s1.pdf]

Supplementary Information:
Tuning-free controller to accurately regulate flow rates in a microfluidic network

Young Jin Heo, Junsu Kang, Min Jun Kim, and Wan Kyun Chung

Supplementary Video S1. Experimental results of valveless flow switching, two laminar interface position control, fine droplet generation and particle manipulation in Figs. 3, 4, 5 and 6.

Text S.1 8 channel network for a case study

In this section, we introduce a more complex microfluidic network (Fig. S.1) than the Y-junction network presented in the paper. This case study that uses the complex network will clarify **1) construction of the steady-state gain matrix, 2) selection of controllable channels, 3) implementation of the proposed controller, and 4) stability of the closed-loop system.**

Text S.1.1 Construction of the steady-state gain matrix

To construct a steady-state gain, the *resistance network* (Fig. S.2) should be used. The network property is as follows:

$y = [Q_1, Q_2, Q_3, Q_4, Q_5, Q_6, Q_7, Q_8]^T$: flow rates at each channel

$u = [p_1, p_2, p_3, p_4]^T$: pressures at each input port

$N_c = 8$: number of channel branches,

$N_n = 4$: number of nodes,

$N_a = 4$: number of input ports,

$N_l = 1$: number of closed loop.

Here, we set the nominal resistances as

$$R_1 = R_4 = R_7 = R_8 = 5[\text{mbar}\cdot\text{min}/\mu\text{L}],$$
$$R_2 = R_3 = R_5 = R_6 = 2[\text{mbar}\cdot\text{min}/\mu\text{L}].$$

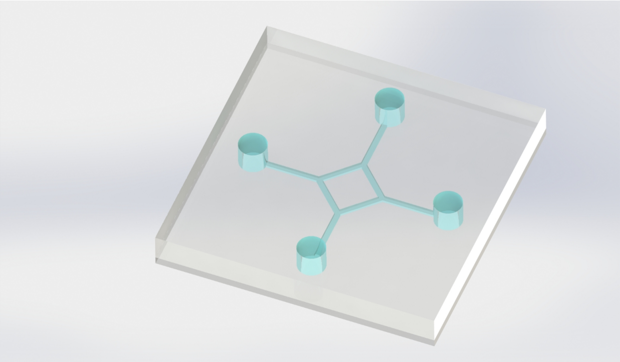

Figure S.1: The eight-channel network used in the case study.

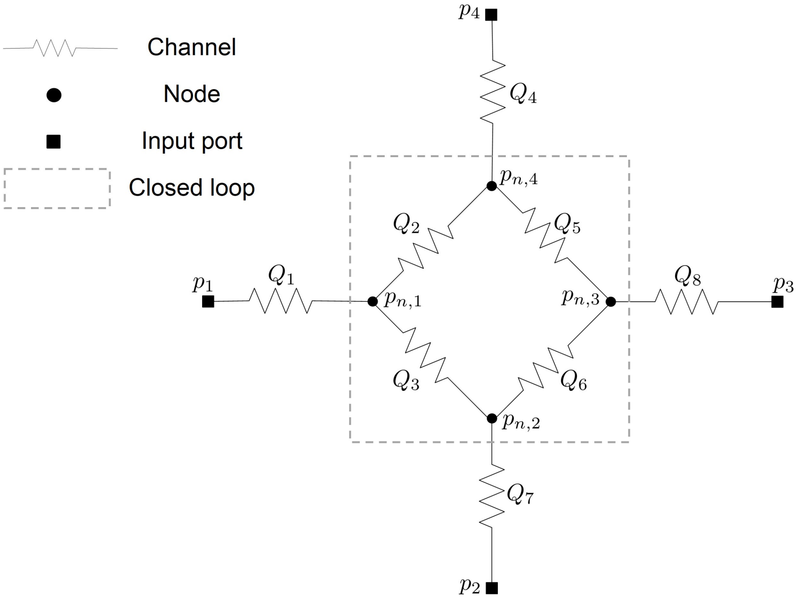

Figure S.2: The resistance network of Fig. S.1

Using (10), A_1 and A_2 are obtained as follows:

$$Q_1 = Q_2 + Q_3 \implies \frac{p_1 - p_{n,1}}{R_1} = \frac{p_{n,1} - p_{n,4}}{R_2} + \frac{p_{n,1} - p_{n,2}}{R_3} \tag{S.1}$$

$$Q_2 = Q_4 + Q_5 \implies \frac{p_{n,1} - p_{n,4}}{R_2} = \frac{p_{n,4} - p_4}{R_4} + \frac{p_{n,4} - p_{n,3}}{R_5} \tag{S.2}$$

$$Q_3 = Q_6 + Q_7 \implies \frac{p_{n,1} - p_{n,2}}{R_3} = \frac{p_{n,2} - p_{n,3}}{R_6} + \frac{p_{n,2} - p_2}{R_7} \tag{S.3}$$

$$Q_5 + Q_6 = Q_8 \implies \frac{p_{n,4} - p_{n,3}}{R_5} + \frac{p_{n,2} - p_{n,3}}{R_6} = \frac{p_{n,3} - p_3}{R_8} \tag{S.4}$$

$$\underbrace{\begin{pmatrix} -\frac{1}{R_1} - \frac{1}{R_2} - \frac{1}{R_3} & \frac{1}{R_3} & 0 & \frac{1}{R_2} \\ \frac{1}{R_2} & 0 & \frac{1}{R_5} & -\frac{1}{R_2} - \frac{1}{R_4} - \frac{1}{R_5} \\ \frac{1}{R_3} & -\frac{1}{R_3} - \frac{1}{R_6} - \frac{1}{R_7} & \frac{1}{R_6} & 0 \\ 0 & \frac{1}{R_6} & -\frac{1}{R_5} - \frac{1}{R_6} - \frac{1}{R_8} & \frac{1}{R_5} \end{pmatrix}}_{A_1} \underbrace{\begin{pmatrix} p_{n,1} \\ p_{n,2} \\ p_{n,3} \\ p_{n,4} \end{pmatrix}}_{p_{node}}$$

$$= \underbrace{\begin{pmatrix} -\frac{1}{R_1} & 0 & 0 & 0 \\ 0 & 0 & 0 & -\frac{1}{R_4} \\ 0 & -\frac{1}{R_7} & 0 & 0 \\ 0 & 0 & -\frac{1}{R_8} & 0 \end{pmatrix}}_{A_2} \underbrace{\begin{pmatrix} p_1 \\ p_2 \\ p_3 \\ p_4 \end{pmatrix}}_{p=u}. \tag{S.5}$$

From (11), A_3 and A_4 also can be obtained as

$$\begin{aligned}
\Delta p_1 &= p_1 - p_{n,1} \\
\Delta p_2 &= p_{n,1} - p_{n,4} \\
\Delta p_3 &= p_{n,1} - p_{n,2} \\
\Delta p_4 &= p_{n,4} - p_4 \\
\Delta p_5 &= p_{n,4} - p_{n,3} \\
\Delta p_6 &= p_{n,2} - p_{n,3} \\
\Delta p_7 &= p_{n,2} - p_2 \\
\Delta p_8 &= p_{n,3} - p_3
\end{aligned}$$

$$\underbrace{\begin{pmatrix} \Delta p_1 \\ \Delta p_2 \\ \Delta p_3 \\ \Delta p_4 \\ \Delta p_5 \\ \Delta p_6 \\ \Delta p_7 \\ \Delta p_8 \end{pmatrix}}_{\Delta p} = \underbrace{\begin{pmatrix} -1 & 0 & 0 & 0 \\ 1 & 0 & 0 & -1 \\ 1 & -1 & 0 & 0 \\ 0 & 0 & 0 & 1 \\ 0 & 0 & -1 & 1 \\ 0 & 1 & -1 & 0 \\ 0 & 1 & 0 & 0 \\ 0 & 0 & 1 & 0 \end{pmatrix}}_{A_3} \underbrace{\begin{pmatrix} p_{n,1} \\ p_{n,2} \\ p_{n,3} \\ p_{n,4} \end{pmatrix}}_{p_{node}} + \underbrace{\begin{pmatrix} 1 & 0 & 0 & 0 \\ 0 & 0 & 0 & 0 \\ 0 & 0 & 0 & 0 \\ 0 & 0 & 0 & -1 \\ 0 & 0 & 0 & 0 \\ 0 & 0 & 0 & 0 \\ 0 & -1 & 0 & 0 \\ 0 & 0 & -1 & 0 \end{pmatrix}}_{A_4} \underbrace{\begin{pmatrix} p_1 \\ p_2 \\ p_3 \\ p_4 \end{pmatrix}}_{p=u} \tag{S.6}$$

The conductance matrix is $B = diag(1/R_1, \cdots, 1/R_8)$ and can be represented as

$$B = \begin{pmatrix} 0.2 & 0 & 0 & 0 & 0 & 0 & 0 & 0 \\ 0 & 0.5 & 0 & 0 & 0 & 0 & 0 & 0 \\ 0 & 0 & 0.5 & 0 & 0 & 0 & 0 & 0 \\ 0 & 0 & 0 & 0.2 & 0 & 0 & 0 & 0 \\ 0 & 0 & 0 & 0 & 0.5 & 0 & 0 & 0 \\ 0 & 0 & 0 & 0 & 0 & 0.5 & 0 & 0 \\ 0 & 0 & 0 & 0 & 0 & 0 & 0.2 & 0 \\ 0 & 0 & 0 & 0 & 0 & 0 & 0 & 0.2 \end{pmatrix} \tag{S.7}$$

Using (14) and substituting the nominal resistance values, the steady-state gain can be obtained as

$$G_{ss} = B(A_3 A_1^{-1} A_2 + A_4) = \begin{pmatrix} 0.1288 & -0.0455 & -0.0379 & -0.0455 \\ 0.0644 & 0.0189 & -0.0189 & -0.0644 \\ 0.0644 & -0.0644 & -0.0189 & 0.0189 \\ 0.0455 & 0.0379 & 0.0455 & -0.1288 \\ 0.0189 & -0.0189 & -0.0644 & 0.0644 \\ 0.0189 & 0.0644 & -0.0644 & -0.0189 \\ 0.0455 & -0.1288 & 0.0455 & 0.0379 \\ 0.0379 & 0.0455 & -0.1288 & 0.0455 \end{pmatrix}. \tag{S.8}$$

Text S.1.2 Simultaneously controllable channels and existence of a right inverse of the steady-state gain matrix

To prove the existence of a right inverse of G_{ss}, we define *controllable channels* in a microfluidic network. All networks (or undirected graphs) that have m branches (edges), n nodes (vertices) and l closed loops have a topology relation $m = n + l - 1$. If a microfluidic network is composed of N_c channels, N_n nodes, N_a input ports and N_l closed loops where vertices are nodes and input ports (i.e., $n = N_n + N_a$), then the topology relation yields $N_c = N_n + N_a + N_l - 1$. This can be rewritten as

$$\pi = N_c - N_n - N_l = N_a - 1 \tag{S.9}$$

where π is a rank of G_{ss} (i.e., $\pi = rank(G_{ss})$). The rank represents 'the maximum number of simultaneously controllable channels' in a microfluidic network. In the Y-junction network, $\pi = 3-1 = 2$, so flow in an arbitrary two of the three channels in the network can be simultaneously controlled, and flow in the other channel is automatically determined by mass conservation (e.g., $Q_1 = Q_2 + Q_3$ in the Y-junction network). The number of controllable channels is two, so three input sources (one inlet and two outlets (Figs. 2, 3, 6), or two inlets and one outlet (Figs. 4, 5)) are required. Here, an outlet that is not selected as a controllable channel can be open to the atmosphere (Fig. 4, 5). Therefore, inlet and outlet should be selected appropriately depending on the purpose of the application.

In matrix G_{ss}, the j-th row concerns the j-th output flow, and the rows of G_{ss} must be reduced to $< \pi$ because the number of controllable channels cannot exceed π. Here, if $\pi < N_a$, then the reduced steady-state gain G_{ss} has a full-row rank and therefore always has a right inverse matrix. If $\pi = N_a$, then G_{ss} is a square and invertible matrix.

In the case study, the maximum number of simultaneously controllable channels is

$$\pi = N_c - N_n - N_l = 8 - 4 - 1 = 3. \tag{S.10}$$

Therefore, three channels of the network can be simultaneously controllable and the matrix G_{ss} always has a right inverse matrix ($\because \pi = 3 < 4 = N_a$).

If we would like to simultaneously control two of the three controllable channels, then

$$\pi = 2 = N_a - 1 \text{ and } N_a = 3, \tag{S.11}$$

so three pressure sources are required.

Text S.1.3 Selection of controllable channels

We can arbitrarily select controllable channels by selectively eliminating rows of G_{ss}. If we want to control Q_2, Q_3 and Q_5, then the 1-st, 4-th, 6-th, 7-th and 8-th rows of G_{ss} should be removed as

$$G_{ss} = \begin{pmatrix} 0.1288 & -0.0455 & -0.0379 & -0.0455 \\ 0.0644 & 0.0189 & -0.0189 & -0.0644 \\ 0.0644 & -0.0644 & -0.0189 & 0.0189 \\ 0.0455 & 0.0379 & 0.0455 & -0.1288 \\ 0.0189 & -0.0189 & -0.0644 & 0.0644 \\ 0.0189 & 0.0644 & -0.0644 & -0.0189 \\ 0.0455 & -0.1288 & 0.0455 & 0.0379 \\ 0.0379 & 0.0455 & -0.1288 & 0.0455 \end{pmatrix} = \begin{pmatrix} 0.0644 & 0.0189 & -0.0189 & -0.0644 \\ 0.0644 & -0.0644 & -0.0189 & 0.0189 \\ 0.0189 & -0.0189 & -0.0644 & 0.0644 \end{pmatrix}, \tag{S.12}$$

where the flow rates inside channel 2, 3, 5 can be independently controlled while the others will be determined by their dependency on the controlled flows. Any three channels in the network can be simultaneously controlled by this method.

By eliminating rows that will not be controlled, G_{ss} becomes a *full row rank matrix* that always has a right inverse matrix. For this case, the right inverse of G_{ss} is

$$G_{ss}^{\dagger} = G_{ss}^T (G_{ss} G_{ss}^T)^{-1} = \begin{pmatrix} 6.0 & 5.5 & 0.5 \\ 6.0 & -11.5 & 5.5 \\ -6.0 & 5.5 & -11.5 \\ -6.0 & 0.5 & 5.5 \end{pmatrix}. \tag{S.13}$$

Text S.1.4 Implementation of the robust controller

Algorithm. S.1 shows the pseudo code for the controller implementation. We set the desired flow rates as $y_{\text{des}} = [30, 20, 10]^T$ (μL/min), and the maximum pressure of the pressure sources as $p_{\max} = [1000, 1000, 1000]^T$ (mbar).

Lines 1 to 3 initialize desired flow rates, the maximum pressure, and the initial control input. From line 4 to 15, the controller turns on. At line 5, flow sensor measures flow rates and the

Algorithm S.1 The pseudo code for the controller implementation

```
Require: G†_ss
 1: y_des = [30, 20, 10]^T;
 2: p_max = [1000, 1000, 1000]^T;
 3: u_c = [0, 0, 0]^T;
 4: while controller on do
 5:     y_m ← GetFlowSensors();
 6:     u_c ← u_c + G†_ss(y_des − y_m);
 7:     for k=1:4 do
 8:         if u_c(k) > p_max(k) then
 9:             u_c(k) = p_max(k);
10:         else if u_c(k) < 0 then
11:             u_c(k) = 0;
12:         end if
13:     end for
14:     PressureRegulator(u_c);
15: end while;
```

measured flow rate is allocated to y_m. At line 6, control input is computed as presented in (4). From line 7 to line 12, the control input is evaluated by the saturation function in (6). At line 14, the computed control input is sent to the pressure regulator to insert control input pressure to the microfluidic chip.

Text S.1.5 Simulation

Numerical simulations of application of the robust controller to the 8-channel network are performed. The desired flow rates and maximum pressures are the same as in Text S.1.4. The real uncertain resistances, unknown capacitances and inductances are set as follows:

$$
\begin{aligned}
&R_{r,1} = R_{r,4} = R_{r,7} = R_{r,8} = 7.4631[\text{mbar}\cdot\text{min}/\mu\text{L}],\\
&R_{r,2} = R_{r,3} = R_{r,5} = R_{r,6} = 3.7315[\text{mbar}\cdot\text{min}/\mu\text{L}],\\
&C_1 = C_4 = C_7 = 0.02, C_2 = C_5 = C_8 = 0.05, C_3 = C_6 = 0.01[\mu\text{L}/\text{mbar}],\\
&L_1 = L_4 = L_7 = 6e-05, L_2 = L_5 = L_8 = 4e-05, L_3 = L_6 = 1e-05[\text{mbar}\cdot\ \text{min}^2/\mu\text{L}].
\end{aligned}
$$

Flow rates of each channel converged simultaneously on the desired flow rates (Fig. S.3a) as a result of rapid adjustment of corresponding pressure inputs (Fig. S.3b). In all channels, the flow rate overshot the target rate, then converged on it by damped oscillations (Fig. S.3c - e); different control frequency shows different transient response. All simulation results are similar to measurement results presented in the paper.

Text S.1.6 Stability of the closed-loop system

Text S.1.6.1 Continuous time case

The stability of the closed-loop system will be evaluated. The transfer matrix function of the nominal system is

$$
G(s) = \begin{pmatrix} G_{11}(s) & G_{12}(s) & G_{13}(s) & G_{14}(s) \\ G_{21}(s) & G_{22}(s) & G_{23}(s) & G_{24}(s) \\ G_{31}(s) & G_{32}(s) & G_{33}(s) & G_{34}(s) \end{pmatrix}, \tag{S.14}
$$

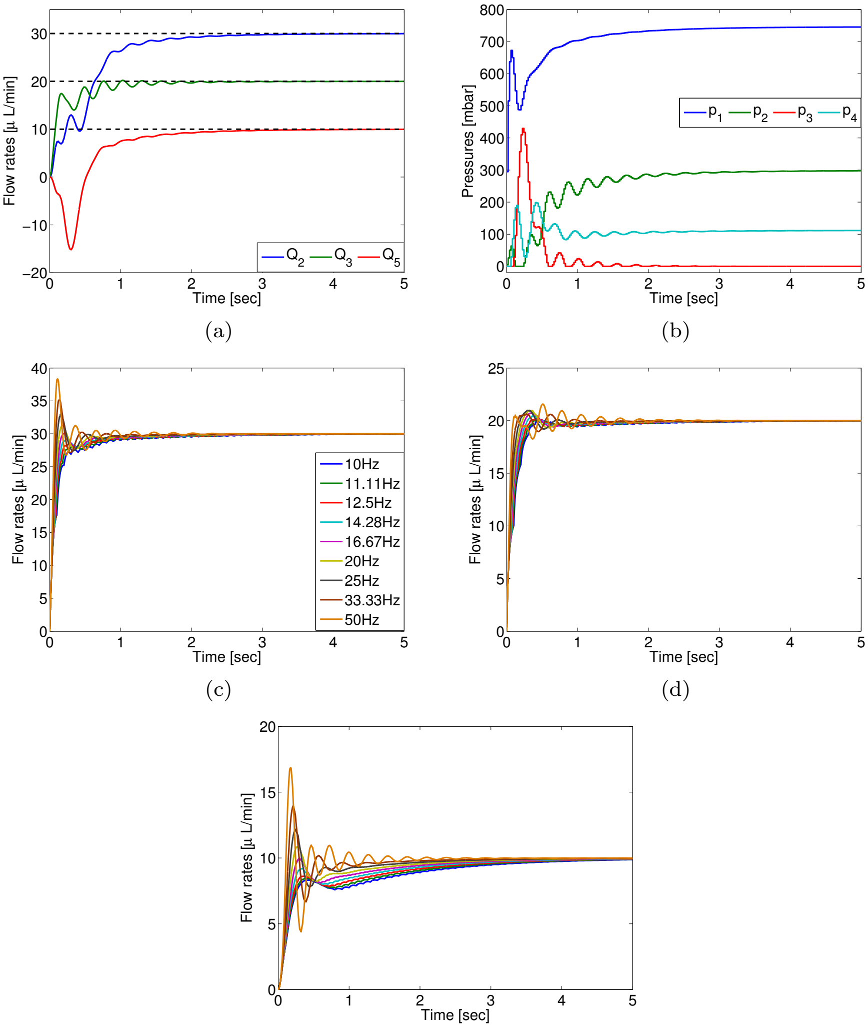

(e)

Figure S.3: **Numerical simulation results in the 8-channel network.** The same strategy performed in the paper is used. The simulation results are similar as the results presented in the paper.

where the transfer matrix function is constructed by synthesizing eight T-models as presented in the paper. The transfer matrix function of the proposed controller is

$$C(s) = \frac{K_{ss}}{s} = \begin{pmatrix} \frac{6.0}{\Delta ts} & \frac{5.5}{\Delta ts} & \frac{0.5}{\Delta ts} \\ \frac{6.0}{\Delta ts} & \frac{-11.5}{\Delta ts} & \frac{5.5}{\Delta ts} \\ \frac{-6.0}{\Delta ts} & \frac{5.5}{\Delta ts} & \frac{-11.5}{\Delta ts} \\ \frac{-6.0}{\Delta ts} & \frac{0.5}{\Delta ts} & \frac{5.5}{\Delta ts} \end{pmatrix} \quad \text{where} \quad K_{ss} = \frac{G_{ss}^{\dagger}}{\Delta t}. \tag{S.15}$$

By constructing a feedback loop, which consists of the controller $C(s)$ in negative feedback with the plant $G(s)$, the transfer matrix function of the closed-loop system is obtained. If poles of the closed-loop system are all negative real, then the closed-loop system is *internally stable*. Moreover, if the system is internally stable, then the origin of state is asymptotically stable [ZDG+]. Here, Δt scales the feedback gain in the controller. Thus, Δt should be bounded in a gain margin of the system. Near the origin, as Δt decreases, the poles go to the right half plane and the closed-loop becomes unstable (Fig. S.4a). In our case study, the closed-loop system becomes unstable when $\Delta t < 0.01$s in the discrete implementation.

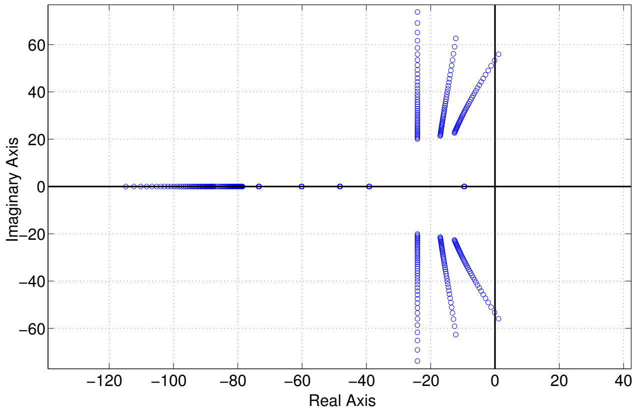

(a)

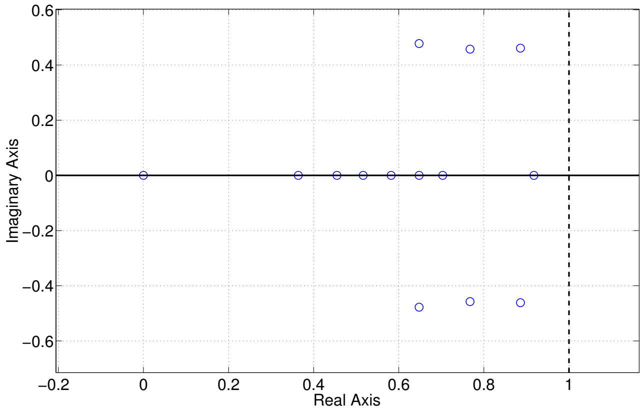

(b)

Figure S.4: **Stability analysis of the closed-loop system.** (a) Closed-loop poles for the continuous case. (b) Closed-loop eigenvalues for the discrete case

Text S.1.6.2 Discrete time case

The closed-loop stability of the discrete system can be directly evaluated. The discrete state-space model of a microfluidic network system is

$$x_{k+1} = \mathcal{A}x_k + \mathcal{B}u_k + v, \tag{S.16}$$
$$y_k = \mathcal{C}x_k + w \tag{S.17}$$

where $\mathcal{A}$, $\mathcal{B}$ and $\mathcal{C}$ are constant matrices with appropriate dimensions; the model obtained by minimal realization of $G(s)$. v and w are unknown constant vectors that can represent constant model uncertainties or slowly-varying process disturbances [DR12]. By substituting the control input into (S.16) and subtracting the previous time-step equation from (S.16) and (S.17), the model can be rewritten as

$$\Delta x_{k+1} = \mathcal{A}\Delta x_k + \mathcal{B}\Delta u_k = \mathcal{A}\Delta x_k - \mathcal{B}G_{ss}^{\dagger}(y_k - y_{\text{des}}), \tag{S.18}$$
$$(\Delta x_k = x_k - x_{k-1}, \Delta u_k = u_k - u_{k-1} = -G_{ss}^{\dagger}(y_k - y_{\text{des}})),$$
$$y_k - y_{\text{des}} = y_{k-1} - y_{\text{des}} + \mathcal{C}\Delta x_k \tag{S.19}$$

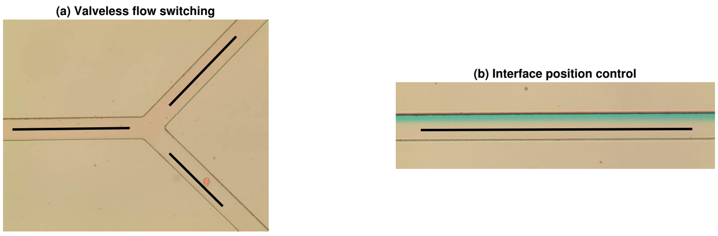

Figure S.5: **Evaluated Lines for Intensity Computation:** Intensity at center of each channel is computed. Black solid lines: location of color intensity measurement in (a) the valveless flow switching experiment and (b) the interface position control experiment.

Therefore, closed-loop dynamics to which the proposed controller is applied can be represented by the augmented state model as

$$\underbrace{\begin{pmatrix} \Delta x_{k+1} \\ y_k - y_{\text{des}} \end{pmatrix}}_{\tilde{x}_{k+1}} = \underbrace{\begin{pmatrix} \mathcal{A} & -\mathcal{B}G_{ss}^{\dagger} \\ \mathcal{C} & I \end{pmatrix}}_{\tilde{\mathcal{A}}} \underbrace{\begin{pmatrix} \Delta x_k \\ y_{k-1} - y_{\text{des}} \end{pmatrix}}_{\tilde{x}_k}. \tag{S.20}$$

If all eigenvalues of $\tilde{\mathcal{A}}$ are < 1, then the closed-loop system is asymptotically stable [Che95] (Fig. S.4b). In conclusion, when the proposed robust controller is applied, the output flow rate converges to the desired output flow rate.

Text S.2 Tuning of transient responses using a single parameter

The transient response (not steady-state response) can be tuned by using a single scalable parameter α to alter the original gain matrix $G_{ss}^{\dagger}$ as follows:

$$G_{ss}^{\dagger} \to \alpha G_{ss}^{\dagger}.$$

The closed-loop transient response increases as α increases, and slows as α decreases. This tuning parameter can be used if fast response is required. Also, as we mentioned in Text S.1.6, the closed-loop response can be unstable in the fast discrete implementation. For this case, α can be used to adjust unstable poles to be stable.

Text S.3 Measurement of image intensity

To measure concentrations of a dye solution, we used image intensity computed using a self-developed image-processing algorithm. The works are in three steps: 1) convert the color image to gray-scale; 2) define the centerlines of each channel that will be evaluated (Fig. S.5); 3) compute the intensity of each line. The mean intensity was used to describe the concentration of dye in the liquid solution.

Text S.4 Analysis method for size distribution of droplets

The droplet size distribution was obtained using a self-developed image-processing algorithm. Droplets generated in the Y-junction network have the same vertical length as the channel width (w), but their horizontal length (L) depended on both flow rates. The image processing algorithm to compute the droplet size (horizontal length) proceeds as follows:

1) Binarizing ('im2bw' function in MATLAB)

Table S.1: Flow rates [μL/min], droplet statistics [μm] and sample sizes in droplet generation experiments.

Experiment	Flow rate		Droplet size	
	Water	Oil	Mean	Standard deviation
1	10	1.25	136.6	1.9
2	10	2.50	213.0	5.9
3	10	5.00	276.8	11.5
4	15	7.50	198.2	4.3
5	15	8.00	219.3	4.7
6	20	5.00	162.9	2.0

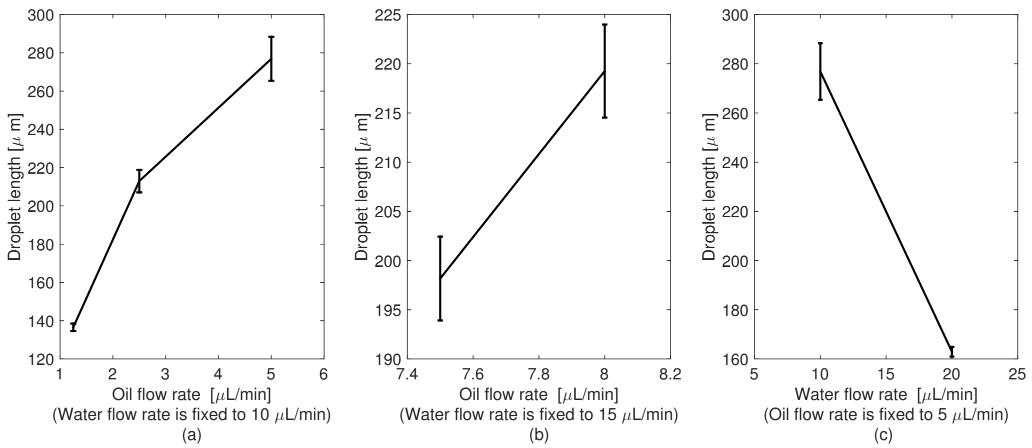

Figure S.6: **Mean droplet size at flow rates of oil and water:** (a) By fixing water flow rate varying oil flow rate. Experiment 1, 2, 3. (b) Experiment 4, 5. (c) Experiment 3, 6. Bars: ± 1 s.d.

2) Filling ('imfill' function in MATLAB)
3) Morphological opening (erosion followed by dilation; 'imerode' and 'imdilate' functions in MATLAB)
4) Blob labeling ('bwlabel' function in MATLAB)
5) Matching each labeled droplet with the prior frame
6) Recording the size until the droplet disappear
7) Averaging the sizes

Theoretical analysis of droplet size with respect to flow rates in a T-junction which is similar to the Y-junction was published previously [GFSW06]. That study identified a scaling law for droplet length (L) with respect to flow rate:

$$L \sim 1 + aQ_{\text{oil}}/Q_{\text{water}} \tag{S.21}$$

In our experiments (Table S.1), L increased as oil flow rate increased (Fig. S.6a, b) and decreased as water flow rate increased (Fig. S.6c). Table. S.1 summarizes all droplet experiments.

References

[Che95] Chi-Tsong Chen. *Linear system theory and design.* Oxford University Press, Inc., 1995.

[DR12] David Di Ruscio. Discrete lq optimal control with integral action: A simple controller on incremental form for mimo systems. *Modeling, Identification and Control*, 33(2):35, 2012.

[GFSW06] Piotr Garstecki, Michael J Fuerstman, Howard A Stone, and George M Whitesides. Formation of droplets and bubbles in a microfluidic t-junctionscaling and mechanism of break-up. *Lab on a Chip*, 6(3):437–446, 2006.

[ZDG+] Kemin Zhou, John Comstock Doyle, Keith Glover, et al. *Robust and optimal control*, volume 40.
